# A single arm trial using passive simulated jogging for blunting acute hyperglycemia

**DOI:** 10.1038/s41598-021-85579-7

**Published:** 2021-03-19

**Authors:** Jose A. Adams, Jose R. Lopez, Veronica Banderas, Marvin A. Sackner

**Affiliations:** 1 Division Neonatology, Mt Sinai Medical Center of Greater Miami, 4300 Alton Road, Miami Beach, FL 33140 USA; 2 Mount Sinai Medical Center of Greater Miami, Miami Beach, FL USA; 3 Sackner Wellness Products LLC, Miami, FL USA

**Keywords:** Metabolism, Translational research, Lifestyle modification

## Abstract

Glycemic fluctuations increase oxidative stress, promote endothelial dysfunction and cardiovascular disease. Reducing glycemic fluctuations is beneficial. We previously reported that a portable motorized passive simulated jogging device, (JD) reduces 24 h glycemic indices in type 2 and non-diabetic subjects. This study evaluates effectiveness and feasibility of JD in blunting large glycemic fluctuation induced by an oral glucose tolerance test (OGTT). The study was performed in 10 adult participants mean age 41.3 ± 13.5 year using interstitial glucose monitor (IG). Each participant fasted for 8 h. followed by an OGTT (Pre-JD), thereafter JD was used for 90 min per day for 7 days, without change to diet or activities of daily living. A repeat OGTT (Post-JD) was performed after completion. The integrated area under the curve (iAUC_2h–4h_) was computed for the OGTT Pre-JD and Post-JD. Seven days of JD blunted the glucose fluctuation produced by OGTT. JD decreased AUC_2h_ by 17 ± 4.7% and iAUC_4h_ by 15 ± 5.9% (p < 0.03). In healthy mostly obese participants 7 days of JD blunts the hyperglycemic response produced by an OGTT. JD may be an adjunct to current glycemic management, it can be applied in different postures for those who cannot (due to physical or cognitive limitations) or will not exercise.

**Trial registration:** *ClinicalTrials.gov* NCT03550105 (08-06-2018).

## Introduction

Spontaneous and postprandial fluctuations/spikes of blood glucose that occur in non-diabetic and type 2 diabetic subjects increase oxidative stress and endothelial dysfunction^1–8^. Such spikes promote accelerated development of atherosclerosis leading to coronary artery disease, stroke, peripheral arterial disease and other vascular pathologies^9–11^. Additionally, hyperglycemia is an independent predictor of death in many acute settings, including acute myocardial infarction, trauma, head injury, stroke and has been shown to occur in the critical care setting in as many as 68% of the patients^12–14^. Therefore, interventions to prevent or minimize these fluctuations should be highly beneficial to health. One such intervention is application of the passive simulated jogging device (Gentle Jogger, Sackner Wellness Products LLC, Miami, FL 33132), a portable, motorized, self-administered, noninvasive movement technology which produces effortless, rapid stepping in place while sitting or lying down. It has been shown to (1) reduce elevated systolic and diastolic blood pressure caused by sitting or lying still^15^, (2) increase short-term heart rate variability^16^ and (3) improve 24 h glycemic indices in type 2 diabetic and non-diabetic participants living at home^17^. In 15 healthy women and 11 men, JD increased oxygen consumption about 15% above the resting seated posture and about 13% above the resting supine posture in moderately obese individuals. None receiving JD reached Metabolic Equivalents (METS) that exceeded 1.5 (a threshold for sedentary behavior)^18^. This current study was carried out to quantitatively evaluate the effectiveness of JD in blunting the major glucose fluctuation induced by an oral glucose tolerance test (OGTT).

## Materials and methods

### Institutional review board approval

This study and its informed consent were approved by Western Institutional Review Board (WIRB) (WIRB, Puyallup, WA 98374-2115) WIRB approved on April 2018, No. 1184829. The study is registered at ClinicalTrials.gov NCT03550105 (06-08-2018) and conducted between September 2018 and May 2019. This study was part of a larger study which evaluated the daily glycemic response, muscle strength and endurance in both healthy volunteers and Type 2 Diabetics. This study was designed as a non-randomized single arm study, each subject served as his or her control. The section of the study reported here are the effects of JD on oral glucose tolerance in healthy volunteers. The inclusion criteria in the current protocol were healthy participants recruited from personal contacts with normal fasting blood glucose and ages between 25 and 85 years. Exclusion criteria included inability to provide informed consent, weight loss surgery, taking anti-diabetic medications, interference with placement of a continuous glucose monitoring device (CGM) during the study period, and lack of compliance to JD protocol. All participants were provided with approved informed consent forms and given the opportunity to ask questions. The CONSORT check list and flow diagram are found in Electronic Supplemental Material File (SEM 1_File).

### Passive simulated jogging device (JD)

The portable JD incorporates microprocessor controlled, DC motorized rapid movements of foot pedals placed within a plastic chassis to repetitively tap against a semi-rigid surface for simulation of locomotion while the subject is seated or lying in a bed. The device which has been previously described and depicted weighs about 4.5 kg with dimensions of 34 × 35 × 10 cm and can be used in supine or seated postures^15^. Its foot pedals alternate between right and left pedal movements to actively lift the forefeet upward about 2.5 cm followed by active downward tapping against a semi-rigid bumper, simulating feet impacting the ground. Each time the moving foot pedals strike the bumper, a small pulse is added to the circulation as a function of pedal speed. The present study protocol used JD speed of ~ 190 steps in place per min.

### Participants

Ten ambulatory individuals without prior history of diabetes who had never taken either insulin or oral diabetic medications were enrolled and gave their informed consent to participate. There were no attempts to modify diet or physical activity, and all participants were told to maintain their normal exercise routine if any. All participants received financial remuneration for their participation. BMI was computed to characterize participants as follows: BMI normal weight 18.5 to 24.9, overweight 25 to 29.9 and obese 30 or more. Demographics are shown in Table 1. Six women and four men constituted the study group. The mean age was 41.3 ± 13.5 year. Using BMI as criteria, three had normal weight, six were overweight, and one was obese.

**Table 1:** Study subject characteristics.

No	Gender	Age	BMI	Glu_Bl_ (mg/dl)
1	M	63	28.9	63
2	M	32	27.5	88
3	F	53	31.8	79
4	F	32	18.5	89
5	F	28	22.9	75
6	M	31	20.3	86
7	F	28	28.2	92
8	F	40	25.4	81
9	F	45	26.7	93
10	M	61	29.6	69
Mean (SD)	6F 4M	41.3 (13.52)	25.9 (4.2)	81.5 (10.0)

This table represent the study subject characteristics; The study subject number (No), gender, age (years), Calculated Body Mass Index (BMI), and baseline glucose values at the beginning of the study prior to the oral glucose tolerance test and use of passive simulated jogging device (Glu_Bl_). Mean and standard deviation (SD) for each column.

### Study protocol

On the first day of study, an interstitial continuous glucose monitor (CGM, FreeStyle Libre Pro, Abbott, Alameda, CA), which provides blood glucose values every 15 min, was fixed over the deltoid area on the non-dominant arm. Participants returned 2 days later after an overnight fast, and a baseline oral glucose tolerance test (OGTT-Pre-JD) was performed using Fisherbrand Oral Glucose Drink (75 g) (Thermo Fisher Scientific, Waltham, MA) which they drank within 2 min.

**Figure 1:**
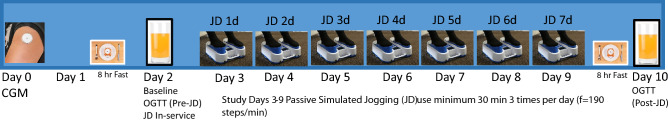
Study protocol. Continuous glucose monitor (CGM) was applied to the non-dominant arm on entering in the study (Day 0). Participants were asked to fast for 8 h prior to the initial baseline oral glucose tolerance test (OGTT) pre passive simulated jogging device (Pre-JD) on Day 2. During the visit participants were instructed on the use of JD. Participants were asked to use JD a minimum of 3 times for 30 min per day for 7 days from day 3 to 9. The evening of day 9 participants were asked to stop use of JD and fast for 8 h. On day 10 a repeat OGTT was performed Post-JD.

Participants were instructed to continue their same diet and physical activity. On day 3 of the study, the participants were taught the operation of JD and requested to use it three times per day for 30 min sessions at 190 pedal steps in place per minute, amounting to greater than 10,000 pedal steps in place per day, for a total of 7 days. To verify compliance with JD use, they were asked to take photographs of the JD monitoring screen at the end of each session daily with a loaned iPhone and to deliver the iPhone to the study coordinator. Participants returned JD after an overnight fast and a second OGTT (OGTT-Post-JD) was administered on the morning of the eighth day. Figure 1 summarizes this protocol.

In a separate study using 5 adult volunteers (3 males and 2 females, age range 50–80 year) we tested whether or not JD can increase nitric oxide (NO) bioavailability using the non-invasive method of downward displacement of the dicrotic notch on the pulse waveform. In brief, descent of the dicrotic notch occurs because NO dilates resistance vessels, thereby delaying pulse wave reflection^19–22^. The dicrotic notch position is computed from the amplitude of the digital pulse wave (a) divided by the height of the dicrotic notch above the end-diastolic level (b) and designated the a/b ratio. Increase of the a/b ratio due to dicrotic notch descent reflects the vasodilator action of NO on resistance vessels. Thus, release of NO causes downward movement of the dicrotic notch in the diastolic limb of the digital pulse, thereby elevating the a/b ratio^23,24^.

### Data analysis

Interstitial glucose values (IG, mg/dl) every 15 min were exported as a text file to an Excel Spreadsheet which were analyzed starting at 8 a.m. for the baseline day and day 8 after 7 days of JD treatment. No data points were extrapolated or deleted from the analysis. Baseline interstitial glucose values for each OGTT were obtained from the mean 1 h IG measurements prior to ingestion of 75 g of glucose beverage; thereafter, IG values were obtained every 15 min over a 4 h period.

Baseline (pre-JD) and 1 to 4 h IG values post ingestion of the glucose beverage were recorded administration and after 7-day treatment with JD. Since these values were not normally distributed, the non-parametric Mann U Whitney test was employed in the statistical analysis (Statistica Software, (Statsoft, TIBCO Software Inc., Palo Alto, CA) Graphs were plotted using GraphPad Prism 8 (GraphPad Software, San Diego, CA). Significant differences between means were taken as p < 0.05. In addition, 2 and 4 h values of each subject’s iAUC_2h_ and iAUC_4h_ were obtained using all 15 min IG values. In order to gauge the magnitude of effect size of the JD on both iAUC_2h_ and iAUC_4h_, we computed *Cohen’s d*. A value greater than or equal to 0.8 denotes a large effect. The primary endpoint of this study was iAUC. Using a 10% change in iAUC, probability of a type 1 error (α = 0.05) and the probability of a type 2 error (ß = 0.15), a sample size n = 10 would yield a power of 0.85. Data are mean ± standard deviation.

### Ethics approval

All procedures performed in studies involving human participants were in accordance with the ethical standards of the institutional and/or national research committee and with the 1964 Helsinki Declaration and its later amendments or comparable ethical standards. The study and its informed consent were approved by Western Institutional Review Board (WIRB) (WIRB, Puyallup, WA 98374-2115) on April 2018, WIRB No. 1184829.

### Consent to participate

Informed consent was obtained from all individual participants included in the study.

### Consent for publication

All authors have reviewed the data and manuscript and endorse its conclusion and consent to its publications.

## Results

**Figure 2:**
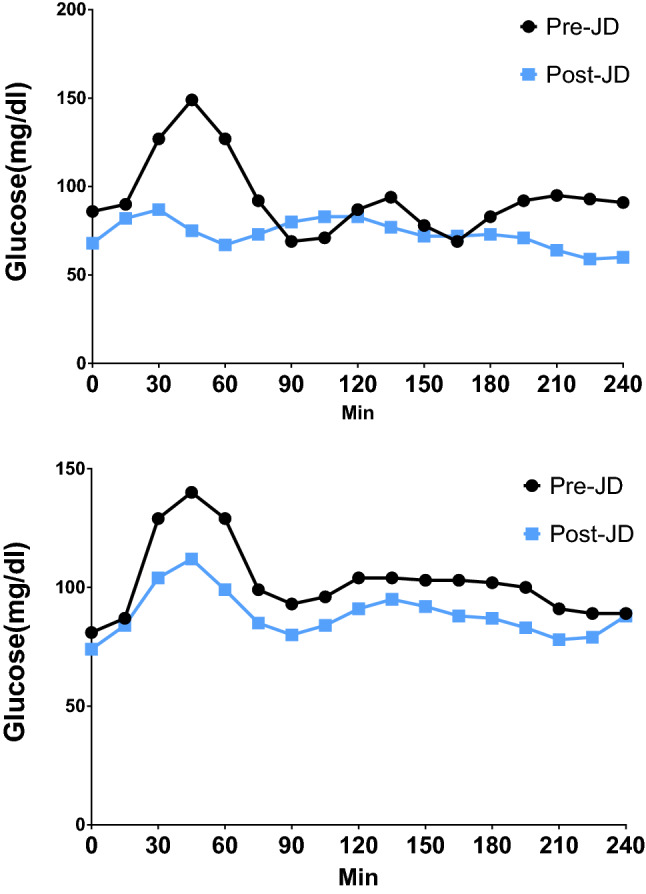
Oral glucose tolerance curves pre and post passive simulated jogging device. Representative 4 h Oral Glucose Tolerance Curves (OGTT) before (Pre-JD) and after 7 days of JD use (Post-JD) from two participants A, B.

Figure 2 depicts IG values over a 4 h period for the OGTT in two representative non-diabetic participants pre and post JD. There is major blunting of the large IG fluctuation associated with glucose ingestion. All ten participants had normal fasting glucose values as measured with CGM, e.g., between 70 and 99 mg/dl both before and after JD administration^25^.

**Figure 3:**
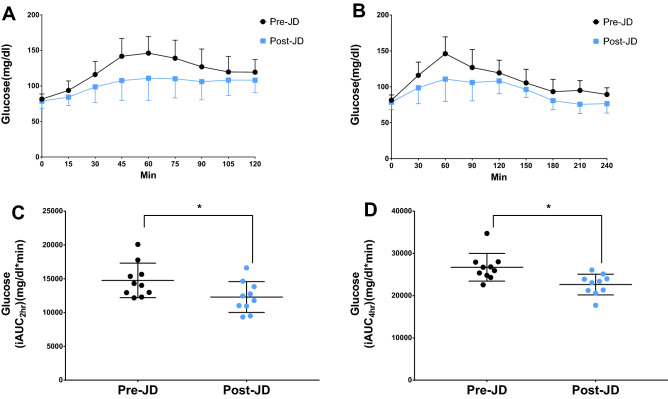
Mean curves and integrated area under the curve of the 2 and 4 h oral glucose tolerance test. Mean and standard deviation for each time point of the 2 h (**A**) and 4 h (**B**) Oral Glucose Tolerance Test (OGTT), before (Pre-JD) and after (Post-JD) 7 days of passive simulated jogging (JD). Mean and standard deviation for the Integrated Area Under Curve (iAUC) of the 2 h (**C**) and 4 h (**D**) OGTT for each subject, before (Pre-JD) and after 7 days of JD use (Post-JD) (*p < 0.005 Pre-JD vs Post-JD).

The 1 and 2 h interstitial glucose values pre and post JD for the OGTT are listed in Table 2. Since the 1 h OGTT plasma glucose value as equated with IG greater than 155 mg/dl is predictive of prediabetes^26,27^, 3 of the 10 participants could be considered prediabetes prior to the administration of JD. Following JD treatment for 7 days, 9 of the 10 participants had normal 1 and 2 h glucose values after OGTT administration. One subject had abnormal (158 mg/dl) 1 h glucose (but markedly decreased from pre-JD value (217 mg/dl), and normal (135 mg/dl) 2 h glucose after OGTT post-JD. Figure 3 shows the values for 2 and 4 h mean curves for all subjects and integrated area under the curve (iAUC_2 and 4h_) of the OGTT pre and post JD. Seven days of JD decreased both the 2 and 4 h iAUC by 17% ± 4.7 and 15% ± 5.9 respectively (p < 0.03 and p < 0.002). The effect size of JD on iAUC_2 and 4h_ was *d* = 1.01 and *d* = 1.41 respectively. There was no significant difference in the time to peak glucose for pre and post JD. Peak glucose during Pre-JD occurred at 63 min (range 45–135 min) and Post-JD 67.5 min (range 30–120 min), (p = 0.72).

**Table 2:** Interstitial glucose values at 1 and 2 h during the oral glucose tolerance test, and area under the curve (iAUC_2h_ and iAUC_4h_) before (Pre-JD) and after 7 days of passive simulated jogging (Post-JD).

No	Pre-JD				Post-JD			
	1 h glucose (mg/dl)	2 h glucose (mg/dl)	iAUC_2h_ (mg/dl min)	iAUC_4h_ (mg/dl min)	1 h glucose (mg/dl)	2 h glucose (mg/dl)	iAUC_2h_ (mg/dl min)	iAUC_4h_ (mg/dl min)
1	135	125	12,945	25,920	114	109	11,783	23,063
2	167	105	15,653	28,020	140	113	13,838	23,895
3	137	110	14,018	25,373	80	98	11,033	21,225
4	137	101	14,310	26,805	115	105	12,458	23,985
5	151	115	15,360	26,730	118	93	12,758	23,385
6	127	87	12,173	22,568	67	83	9338	17,730
7	217	127	20,078	27,930	158	135	16,613	26,070
8	129	104	12,983	24,810	99	91	10,958	21,330
9	168	170	17,768	34,733	144	128	14,640	25,118
10	94	150	12,293	24,300	73	127	9503	20,573
Mean (SD)	146.2 (32.7)	119.4 (24.8)	14,758 (2549)	26,719 (3276)	110.8 (31.1)	108.2 (17.5)	12,292 (2284)*	22,637 (2452)*

Peak one and two hour interstitial glucose values [1 h, 2 h Glucose (mg/dl)] and integrated 2 h and 4 h Areas Under the Curve (iAUC_2h,_ iAUC_4h_) during the oral glucose tolerance test, before (Pre-JD) and after 7 days of passive simulated jogging (Post-JD) for each study subject (No). Mean and standard deviation (SD) for each column.

*p < 0.03 (pre vs post JD).

**Figure 4:**
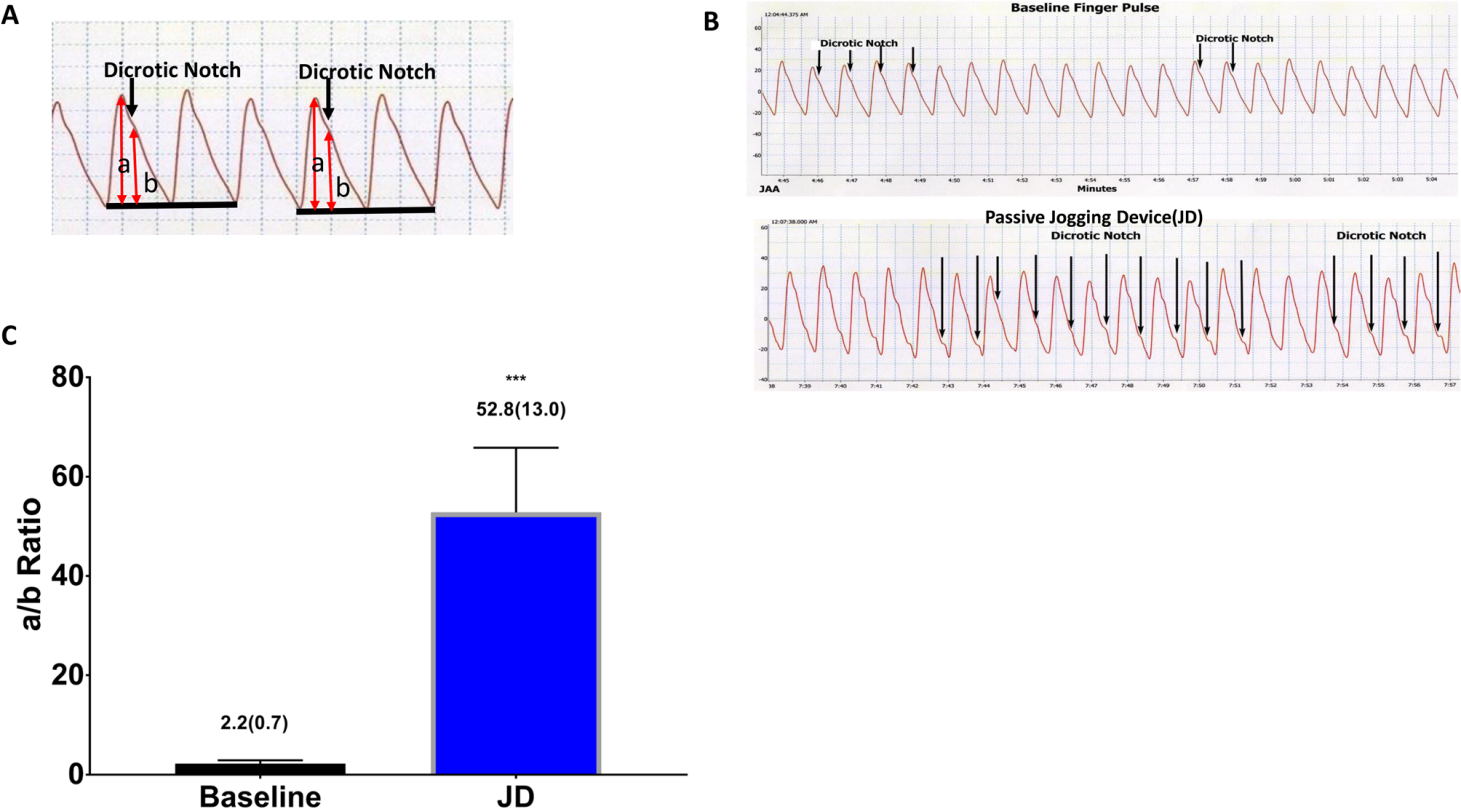
Passive Jogging Device (JD) increases Nitric Oxide (NO) Bioavailability. (**A**) Representative Plethysmographic arterial pulse waveform at baseline denoting the position of the dicrotic notch and the a and b position on the pulse waveform. (**B**) representative tracings of one subjects descent of the dicrotic notch at baseline and during use of JD device (**C**) JD in seated posture increased a/b ratio 24-fold from baseline values, confirming that JD increases NO bioavailability in human subjects. (***p < 0.001 Baseline vs. JD) [MEAN (SD)].

The effects of JD on NO bioavilabity were determined in seated at rest (baseline) and during JD, using the a/b ratio. Figure 4A,B show the measurement of a/b on a plethysmographic waveform. JD increased a/b ratio by 24-fold from baseline values, confirming that JD increases NO bioavailability in human subjects Fig. 4C.

## Discussion

The current study carried out in normal participants extends previous observations in which JD diminished daily glycemic variability in non-diabetic and diabetic patients^17^. JD reduced OGTT iAUC_2h_ 17% ± 4.7 in normal participants by suppressing the amplified glycemic fluctuation produced by OGTT. The latter has a physiologically relevant large effect size of *Cohen’s d* = 1.01^28^. JD by producing passive endothelial pulsatile shear stress, like its predicate device, the motion platform (Whole Body Periodic Acceleration (WBPA) aka pGz), and, aerobic exercise increase bioavailability of nitric oxide^23,29–31^. Such activity is also heightened in fidgeting, which consists of tapping 220–290 times a minute compared to ~ 190 taps per minute utilized during operation of JD^32^. It is noteworthy that Flamenco dancers in performance average about 1000 heel taps per minute but no clinical studies on their cardiovascular health or glucose metabolism have yet been undertaken.

In mostly overweight healthy subjects enrolled in the current study, the high glycemic fluctuation produced by ingestion of glucose for the OGTT was significantly dampened by daily JD usage over a 7-day period. The second OGTT was administered at least 8–14 h following the last JD treatment. Compared to baseline, iAUC_2h_ was reduced by 17% presumably as a result of the action of nitric oxide from upregulation of eNOS and nNOS in skeletal muscle which facilitates blood glucose transport to skeletal muscle^33–35^.

Since exercise and WBPA (predicate device of JD) increase bioavailability of nitric oxide by activation of eNOS and nNOS through increased shear stress^23,24,29–31^, the effects of exercise in reported studies of healthy subjects and prediabetic individuals using iAUC_2h_ from a OGTT after ingestion of 75 g glucose were reviewed for comparison to JD. Such studies included 9–14 normal participants and 26 prediabetics. Daily exercise protocols which were not standardized were performed for 5–14 days and the OGTT obtained 14–24 h following the last exercise procedure for the normal subjects and 8–10 h after the last exercise bout for the prediabetics. In three reports in normal subjects, exercise reduced iAUC_2h_ 5, 13, and 30%^36–38^. In one report of normal subjects and a report of prediabetics, exercise did not change iAUC_2h_^39,40^. In a different study 31 healthy normal weight participants were stratified into self-reported physical activity volume and intensity and received and oral glucose tolerance test (50 g glucose). They found a statistically significant decrease in iAUC with increasing level of physical activity (very active > 60 min/day, high intensity group)^41^. Although these data suggest that suppression of large glycemic excursions as produced in a OGTT might be responsive to exercise and JD, clearly more data are needed for a definitive conclusion.

There are limitations to the present study which must be acknowledged. This study was not designed to specifically determine the mechanisms by with JD blunts hyperglycemia, but some useful information can be gleaned from the data. JD increases NO bioavailability, via endothelial derived nitric oxide (eNO). NO derived from eNO, has been shown to increase insulin secretion, improve insulin signaling and sensitivity, increase peripheral glucose uptake, and decrease hepatic glucose output^42^. It is plausible that JD blunts hyperglycemia thru any of these mechanisms. Alternatively, JD could also stimulate passive stretching, the latter has been known to activate muscle glucose uptake through stretch-stimulated glucose uptake, which does not appear to be endothelial nitric oxide synthase (eNOS) or neuronal NOS (nNOS) dependent since passive stretch failed to increase NOS activity above resting levels^43^.

Changes of IG using the FreeStyle Libre Glucose Monitoring System during OGTT in the current trial and plasma glucose values and iAUC were considered equivalent although the absolute values of plasma glucose have been reported about 11% lower than the IG values^44^. Application of CGM during a home trial renders it feasible to use whereas repeated venipunctures for measurements of plasma glucose are impractical. In our initial study of 24 h glycemic variability over 7 days carried out in 11 healthy subjects, %CV was significantly lower between baseline and JD on most days indicating consistency of JD suppressing IG fluctuations during free living^17^. The small sample size in this study precludes us from making population wide generalizable comments, however the sample size in this study is similar to the previously cited studies, and JD induced a significant physiologically effect^34,37–39^. We are also unable to comment on the effects of extended use of JD beyond 7 days, anecdotal data suggests continued effects while JD is utilized in both diabetics and non-diabetics^17^. The latter is relevant since recent large data base analysis of long-term glycemic variability (glycemic fluctuations over months to years) showed that increased long-term glycemic variability is associated with increase cardiovascular disease and mortality in nondiabetics^45^.

The findings of this study are clinically relevant. Hyperglycemia is a common occurrence in the intensive care unit (ICU), is associated with worse outcomes in both adults and children, in both diabetics and non-diabetics, and an independent predictor of in hospital mortality in critically ill patients^12–14,46,47^. Further, acute increase in glycemia is accompanied by a large surge of inflammatory mediators, which worsens severity of disease^48^. Therefore, an intervention such as the JD which has been shown to decrease 24 h glycemic indices in diabetics and non-diabetics, and to markedly reduce the large post prandial glucose spike, may be an adjunct in the glycemic control of critically ill patients which requires further studies.

## Conclusion

The current trial in healthy mostly overweight participants shows that 7 days of JD significantly blunts the hyperglycemia response produced by an OGTT. JD is a simple to use device which can be used in various postures for those individuals which cannot (due to physical or cognitive limitations) or will not exercise.

## Supplementary information

Supplementary Information 1.

## Data Availability

The datasets generated during and/or analyzed during the current study are available from the corresponding author on reasonable request.
